# The Registration of Nursing Homes

**Published:** 1907-12-14

**Authors:** 


### Nursing Administration.

### THE REGISTRATION OF NURSING HOMES.

### V.—A SUGGESTION.

It is a very interesting circumstance that all the difficulties which have assailed the nursing profession in matters of organisation have been experienced already by the educationalists in their endeavours to regulate the profession of teaching. The need for the registration of teachers was no less pressing than that for the registraton of nurses. It has been tried and it has failed. But in another direction the educationalists have been more successful, and may well serve to point the way for imitation. The monopoly held forty years ago by thoroughly incompetent and mercenary principals of girls' schools was an even graver public danger than is presented by the monopoly of nursing homes under private management to-day. Public opinion was still blind to the futility of the average Young Ladies' Seminary and the law was absolutely indifferent. The matter was settled in the end, as many matters in England are settled, by the formation of public companies charged with the duty of superseding these schools under illiterate private management and replacing them by high schools under the charge in each locality of a representative committee. The subscribers to these companies, for there was soon more than one, were people interested in education who looked merely for a fair return for their money, but there was no design at any time of making more than five per cent. on the capital invested. Any profits over and above this amount was to be bestowed on improving or cheapening the education provided in the establishments under the companies' control. By means of capital thus entrusted to the direction of a few business people, who were keenly interested in the cause of education, the whole aspect of female education was altered within the space of a few years.

The moment is ripe for the establishment in the interests of the medical and nursing profession, and with a view to the safety and comfort of the public, of companies of a similar character charged with the establishment of nursing homes in every town where they are needed, and in various parts of London. It can hardly be doubted that a company started with the aim of establishing and controlling nursing homes in the interests of the public would meet with instantaneous support. "Philanthropy at five per cent.," as such enterprises are sometimes called, is very often not only the best kind of philanthropy, but the best kind of finance. It attracts the support of real enthusiasts. The establishment of Pay Hospitals, for such they would really be, would need to be begun with caution, and to be attempted in the first instance solely in localities where the need was most urgent. An influential committee would be needed to watch over the interests of each establishment, to choose the superintendent, and regulate the fees to be paid. Such a committee, knowing the special requirements of its own neighbourhood, would be able to apportion the accommodation so that several distinct classes of persons could be received, it being always understood that the basis of the undertaking would be to make each separate Home pay its own way, and secure a dividend of five per cent. to the shareholders on the capital subscribed. One after another of the existing nursing homes in the market could thus be acquired, and either remodelled and continued on existing sites or transferred to better premises. It goes without saying that good private nursing homes would still continue to exist, just as good private schools for girls are now abundant, owing all their prosperity to healthy competition. For the existence of numerous excellent pay hospitals under public control would tend to raise the standard, and to render such abuses as now flourish unchecked all round us a thing of the past. The security conferred by public management would tell instantaneously upon the whole character of the nursing home. Responsible business men would lend to the choice of a site for the building on which their dividends depended some of the care and forethought demanded of the hospital committee. They would tolerate nothing cramped, insanitary, or furtive for the sake of saving a few pounds. They would know that such economies are poor business, and that in enterprises which are meant to pay nothing but the best can be tolerated. They could be trusted to engage the best nurses obtainable for the work, and it is probable that they would be prepared to treat their staff liberally. That the public would eagerly take advantage of such publicly controlled nursing homes may be considered an admitted fact. We have only to inquire whether the medical profession would welcome them, and whether the required dividend would be forthcoming. As regards the medical profession, it may be safely assumed that such homes would be received with thankfulness, provided always that the rights of individual practitioners were rigidly safeguarded, and it would be part of any enterprise governed by business men to see that was done. But would the nursing home conducted on these lines pay, even to the modest extent we have indicated? We believe that it would, and we can point to the continued prosperity of Fitzroy House in evidence of the fact that under good management such homes can undoubtedly be conducted at a profit. The difficulty about the profits of the nursing home is that the proprietor is not satisfied with a fair percentage on the outlay. Let it be supposed that a nurse pays £1,000 for the purchase and setting-up of a home. Is she content to draw no more than a bare £50 as her share of the interest accruing, after reckoning off the value of her services as superintendent? Does she not far more often enlarge her personal expenditure to suit with the size of her house, and reckoning all in the expenses of upkeep, run herself into difficulties and debt for want of any clear understanding of what she has a right to expect. It is the introduction of business principles into the affairs of the nursing home which is needed to make such establishments what they certainly are seldom at the present time, a complete business success.

